# Colonic varices treated with embolization after pancreatoduodenectomy with portal vein resection: a case report

**DOI:** 10.1186/s40792-020-00888-9

**Published:** 2020-06-03

**Authors:** Shota Kuwabara, Joe Matsumoto, Hiroyasu Tojima, Hideyuki Wada, Kohei Kato, Yukiko Tabata, Masaomi Ichinokawa, Tatsuya Yoshioka, Katsuhiko Murakawa, Atsushi Ikeda, Setsuyuki Ohtake, Koichi Ono

**Affiliations:** Department of Surgery, Obihiro Kosei General Hospital, West 14 South 10, Obihiro, Hokkaido 080-0024 Japan

**Keywords:** Pancreatic cancer, Pancreatoduodenectomy, Portal vein resection, Left-sided portal hypertension, Colonic varices, Interventional radiology, Embolization

## Abstract

**Background:**

Pancreatoduodenectomy with resection of the portal vein or superior mesenteric vein confluence has been safely performed in patients with pancreatic head cancer associated with infiltration of the portal vein or superior mesenteric vein. In recent years, left-sided portal hypertension, a late postoperative complication, has received focus owing to increased long-term survival with advances in chemotherapy. Left-sided hypertension may sometimes cause fatal gastrointestinal bleeding because of the rupture of gastrointestinal varices. Here, we present a case of colonic varices caused by left-sided portal hypertension after pancreatoduodenectomy with portal vein resection.

**Case presentation:**

A 69-year-old man diagnosed with pancreatic head cancer was referred to our department for surgery after undergoing chemotherapy with nine courses of gemcitabine and nab-paclitaxel. Computed tomography showed a mass 25 mm in diameter and in contact with the portal vein. He had undergone subtotal stomach-preserving pancreatoduodenectomy with portal vein resection. Four centimeters of the portal vein had been resected, and end-to-end anastomosis was performed without splenic vein reconstruction. We had to completely resect the right colic vein, accessary right colic vein, and middle colic vein due to tumor invasion. The pathological diagnosis was ypT3, ypN1a, ypM0, and ypStageIIB, and he was administered TS-1 as postoperative adjuvant chemotherapy. Seven months after therapeutic radical surgery, he presented with melena with progressive anemia. Computed tomography revealed transverse colonic varices. He was offered interventional radiology. Trans-splenic arterial splenic venography showed that transverse colonic varices had developed as collateral circulation of the splenic vein and inferior mesenteric vein system. An embolic substance was injected into the transverse colonic varices, which halted the progression of the anemia caused by melena. Fifteen months after therapeutic radical surgery, local recurrence of the tumor occurred; he died 28 months after the surgery.

**Conclusions:**

When subtotal stomach-preserving pancreatoduodenectomy with portal vein resection is performed without splenic vein reconstruction, colonic varices may result from left-sided portal hypertension. Interventional radiology is an effective treatment for gastrointestinal bleeding due to colonic varices, but it is important to be observant for colonic necrosis and new varices.

## Background

Pancreatoduodenectomy (PD) with resection of the portal vein (PV) or superior mesenteric vein (SMV) confluence has been safely performed in patients with pancreatic head cancer associated with infiltration of the PV or SMV. There are reports demonstrating that splenic vein (SpV) reconstruction is unnecessary, but there is no consensus on this [1–3]. In recent years, left-sided portal hypertension (LPH), a late postoperative complication, has received focus owing to increased long-term survival with advances in chemotherapy. LPH may sometimes cause fatal gastrointestinal bleeding because of rupture of gastrointestinal varices [4]. Here, we present a case of colonic varices caused by LPH after PD with PV resection (PVR).

## Case presentation

**Fig. 1:**
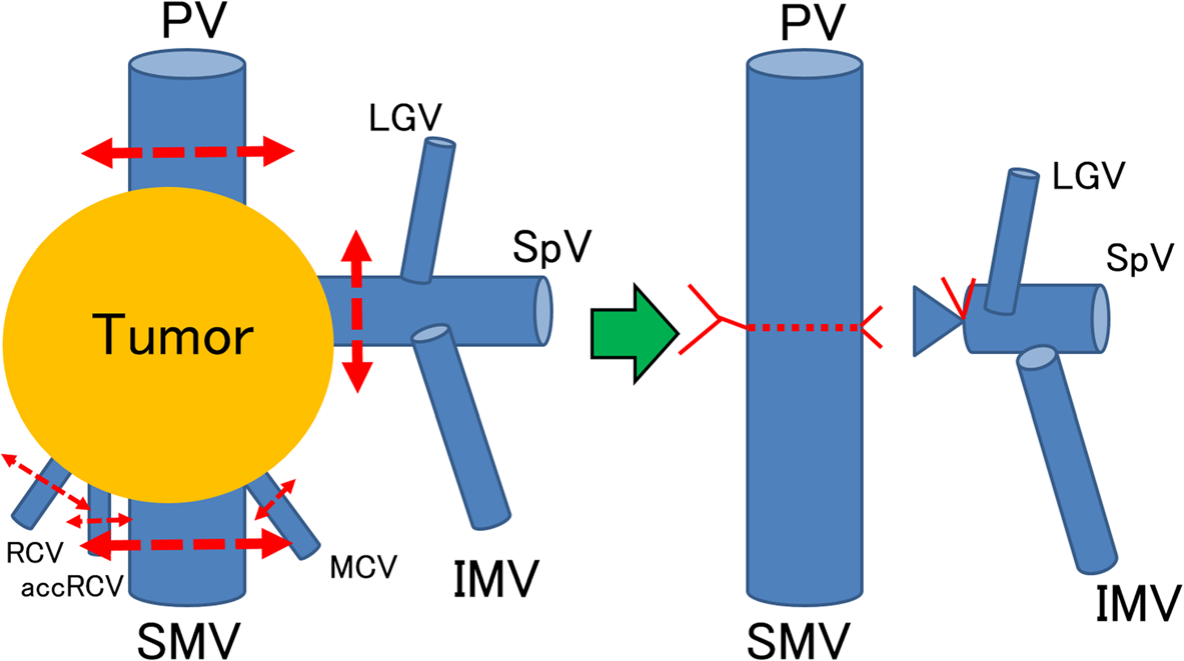
Schema of the operation. The schema of the operation is depicted. SSPPD with PVR was performed. PV had 4 cm of its length resected, and end-to-end anastomosis was performed without reconstructing the SpV. RCV, accRCV, and MCV which received reflux directly from the PV-SMV were all resected due to tumor invasion. PV, portal vein; SMV, superior mesenteric vein; RCV, right colic vein; accRCV, accessary right colic vein; MCV, middle colic vein; LGV, left gastric vein; SpV, splenic vein; IMV, inferior mesenteric vein

**Fig. 2:**
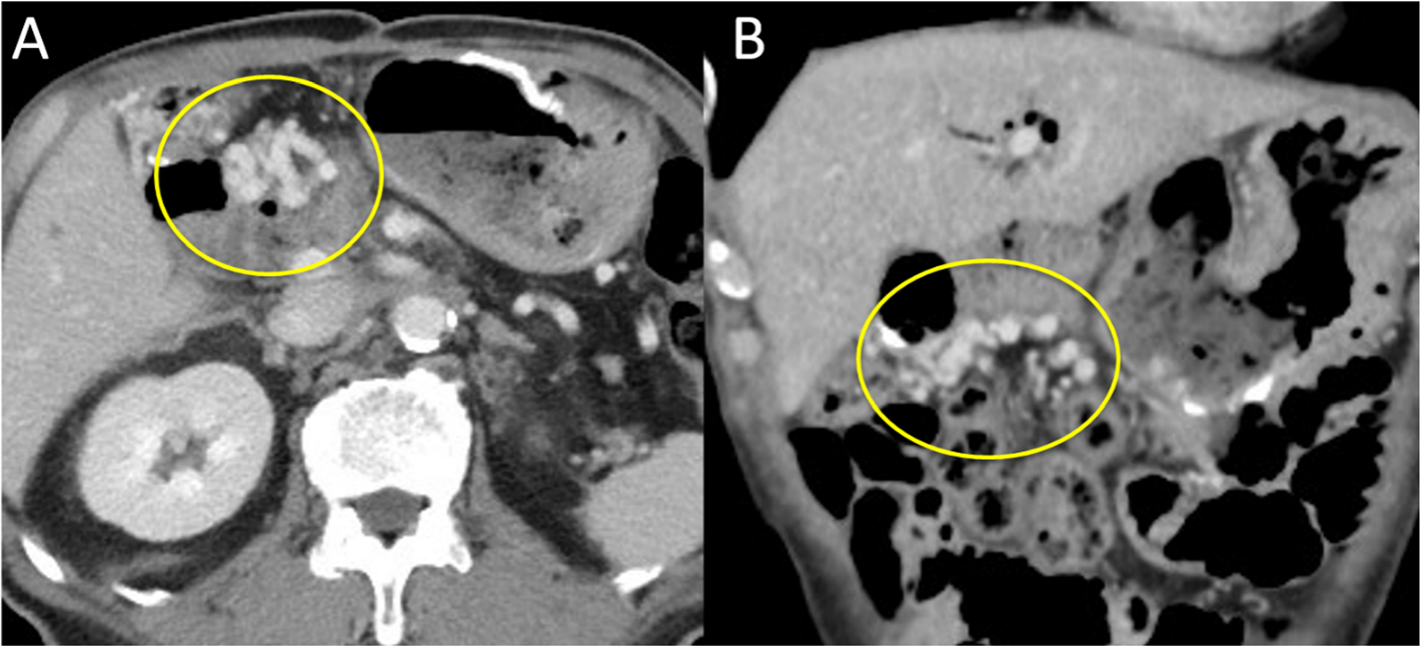
Findings of enhanced computed tomography (CT). **a** Axial imaging. **b** Coronal imaging. CT scan did not show obstruction or stenosis of the reconstructed PV, but it revealed transverse colonic varices (yellow circles)

**Fig. 3:**
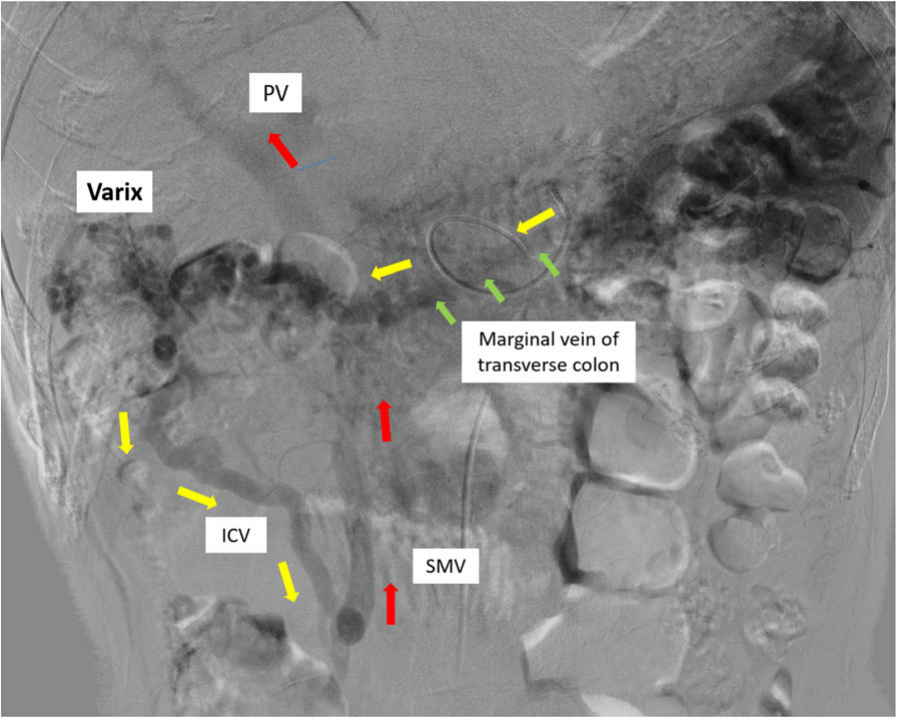
Findings of trans-splenic arterial splenic venography. A route (red arrow) was formed from the marginal vein of the transverse colon (green arrow), via the ICV, into the superior mesenteric vein (yellow allow) and back to the portal vein. PV, portal vein; SMV, superior mesenteric vein; ICV, ileocolic vein

A 69-year-old man was referred to our gastroenterology department because of worsening diabetes. Computed tomography (CT) revealed a mass measuring 22 mm in diameter, in contact with the PV in the pancreatic head. It was identified as an adenocarcinoma by endoscopic ultrasonography (EUS)-guided fine needle aspiration (FNA). He was diagnosed with pancreatic head cancer, but he was not offered therapeutic radical resection because the tumor had invaded the PV. He was referred to our department again for surgery after receiving chemotherapy with nine courses of gemcitabine and nab-paclitaxel. Although the CT scan showed a mass measuring 25 mm in diameter and still in contact with the PV, we judged that the mass was resectable and he had subtotal stomach-preserving pancreatoduodenectomy (SSPPD) with PVR 11 months later. The PV had 4 cm of its length resected, and end-to-end anastomosis was performed without reconstruction of the SpV. We had to completely resect the right colic vein (RCV), accessary right colic vein (accRCV), and middle colic vein (MCV) which received reflux directly from the PV-SMV due to tumor invasion (Fig. 1). The pathological diagnosis was ypT3, ypN1a, ypM0, and ypStageIIB, and he was administered TS-1 as postoperative adjuvant chemotherapy. Seven months after therapeutic radical surgery, he developed black stools with progressive anemia. CT did not show obstruction or stenosis of the reconstructed PV, but it revealed transverse colonic varices (Fig. 2). The diameter of the spleen increased preoperatively from 67 to 74 mm, but there were no symptoms of hypersplenism such as thrombocytopenia. He was offered interventional radiology (IVR). Trans-splenic arterial splenic venography showed that transverse colonic varices had developed as collateral circulation of the SpV and inferior mesenteric vein (IMV) system. A balloon catheter was inserted into the splenic artery via the right inguinal arterial approach, and a microcatheter for embolization was introduced retrograde to the colonic varices via the intrahepatic PV. An embolic substance was injected into the transverse colonic varices (Fig. 3). This halted the progression of the anemia caused by melena. During follow-up after IVR, esophagogastroduodenoscopy and colonoscopy did not show any gastrointestinal varices. Fifteen months after therapeutic radical surgery, local recurrence of the tumor occurred, and he died 28 months after therapeutic radical surgery and 39 months after diagnosis.

## Discussion

PD with combined PVR can be performed safely with improved surgical techniques [5], and recently, there have been reports that patients undergoing PVR for pancreatic head cancer may have the same survival rate as patients who do not undergo PVR [6]. Advancement of multidisciplinary treatment combining chemotherapy, radiation therapy, and surgical treatment has improved long-term survival in patients with locally advanced pancreatic cancer, and LPH is commonly observed as a late complication. In general, LPH is thought to cause gastrointestinal varices and splenomegaly with thrombocytopenia due to loss of splenic blood reflux because of obstruction of the SpV by the malignant tumor or pancreatitis, despite normal liver function and portal venous blood flow. Recently, it has been shown that LPH can also occur when the PV-SMV confluence is resected together with PD, as seen in this case. Two routes are commonly responsible for collateral circulation: a route from the SpV to the stomach via the omentum, and then to the PV via the left gastric vein (LGV), and another route from the SpV to the superior vena cava via the azygos vein, after flowing into the esophagus. These routes may lead to gastric varices and esophageal varices, respectively. Another route from the SpV into the PV via the peripancreatic or pancreatojejunostomy may result in pancreatic varices. In addition, there may be another route from the SpV to the SMV and/or PV via the IMV via the marginal vein of the left-sided or transverse colon and/or the right-sided colon, which may cause colonic varices [7]. In addition to these four routes, there are two non-varicose routes: splenic-colonic collateral circulation (arc of Barkow) and physiological splenic renal shunt [1]. Regarding the incidence of colonic varices, a previous study involving 43 cases that underwent PD with PVR reported that colonic varices developed in 27 cases (63%) and 3 cases required treatment [4]. In the index case, RCV, accRCV, and MCV had to be resected due to tumor invasion and the preserved LGV and IMV flowed into the SpV, leading to inadequate venous return, which might have led to the formation of colonic varices. Besides, the mid-part of the omentum was divided during SSPPD in the present case, which can preserve the collateral route in the mesocolon via the marginal vessels of the transverse colon. A route may have formed from the marginal vein of the transverse colon to the SMV via the ileocolic vein (ICV), resulting in colonic varices (Fig. 3).

Some have considered reconstructing the SpV to prevent LPH. Although there have been reports that it is important to preserve the flow of the LGV and MCV directly into the PV-SMV when SpV reconstruction is unnecessary [1–3], it may not be possible when tumor invasion necessitates resection, as in this case. There is also a report that the simultaneous resection of the splenic artery can prevent the occurrence of varices due to LPH, but in this case, there was a risk of severe complications such as splenic infarction and residual gastric blood flow disorder [7]. On the other hand, there are reports that SpV-PV anastomosis is useful for preventing gastrointestinal bleeding [8], and a systemic shunt by SpV-left renal vein anastomosis is useful [9] when SpV reconstruction is necessary. Theoretically, SpV-inferior vena cava anastomosis is also conceivable, but the evidence is limited. When performing vascular anastomosis reconstruction, organ failure from insufficient blood flow caused by anastomotic stenosis or thrombus formation may pose a serious problem. In particular, PV thrombus due to tortuous or bending SpV-PV anastomosis can be fatal. In addition, if the length of anastomosis is inadequate, a biological vascular graft or an artificial vascular graft may be required as it affects postoperative vascular graft patency [7]. In any case, vascular anastomosis reconstruction requires advanced surgical techniques, so it must be performed by well-experienced surgeons in appropriate facilities. However, if anastomosis is anatomically challenging, careful preoperative evaluation is required to determine whether reconstruction is possible. Prophylactic splenectomy is effective in reducing left PV pressure, but simultaneous resection increases the risk of pancreatic fistula, intraoperative bleeding, and severe postoperative infection. In the present case, SpV reconstruction (SpV-PV or SpV-left renal vein anastomosis) may possibly have prevented colonic varices.

For varices, local treatment with IVR may be useful, but splenectomy or splenic embolization may be considered if control of bleeding varices is difficult [10]. In the present case, embolization by IVR was preferred for local treatment of colonic varices due to LPH. When the marginal vein of the colon is obstructed by embolization of varices, colonic necrosis may occur due to congestion, and surgical colectomy would be necessary. Gastrointestinal bleeding may be caused by the occurrence of new varices, so careful follow-up by gastrointestinal endoscopy is required.

## Conclusions

When SSPPD with PVR is performed without reconstruction of the SpV, colonic varices may result from LPH. IVR is one of the effective treatments for gastrointestinal bleeding due to colonic varices, but it is important to be observant for colonic necrosis and new varices.

## Data Availability

The datasets supporting the conclusions of this article are included within the article.

## Abbreviations

LPH: Left-sided portal hypertension
SSPPD: Subtotal stomach-preserving pancreatoduodenectomy
PVR: Portal vein resection
IVR: Interventional radiology
